# Cytoprotective Effect of Vitamin D on Doxorubicin-Induced Cardiac Toxicity in Triple Negative Breast Cancer

**DOI:** 10.3390/ijms22147439

**Published:** 2021-07-12

**Authors:** Kevin J Lee, Griffin Wright, Hannah Bryant, Leigh Ann Wiggins, Valeria L. Dal Zotto, Michele Schuler, Christopher Malozzi, Michael V Cohen, Natalie R Gassman

**Affiliations:** 1 Department of Physiology and Cell Biology, University of South Alabama College of Medicine, Mobile, AL 36688, USA; gmw1821@jagmail.southalabama.edu (G.W.); mcohen@southalabama.edu (M.V.C.); 2 Department of Comparative Medicine, University of South Alabama College of Medicine, Mobile, AL 36688, USA; hbryant@southalabama.edu (H.B.); lawiggins@southalabama.edu (L.A.W.); mschuler@southalabama.edu (M.S.); 3 Department of Pathology and Laboratory Medicine, Brody School of Medicine, East Carolina University, Greenville, NC 27834, USA; dalzottov21@ecu.edu; 4 Department of Microbiology and Immunology, University of South Alabama College of Medicine, Mobile, AL 36688, USA; 5 Department of Medicine, University of South Alabama College of Medicine, Mobile, AL 36688, USA; cmalozzi@health.southalabama.edu

**Keywords:** antioxidant, oncology, cardiology, reactive oxygen species, chemotherapy

## Abstract

Background: Doxorubicin (Dox) is a first-line treatment for triple negative breast cancer (TNBC), but its use may be limited by its cardiotoxicity mediated by the production of reactive oxygen species. We evaluated whether vitamin D may prevent Dox-induced cardiotoxicity in a mouse TNBC model. Methods: Female Balb/c mice received rodent chow with vitamin D_3_ (1500 IU/kg; vehicle) or chow supplemented with additional vitamin D_3_ (total, 11,500 IU/kg). the mice were inoculated with TNBC tumors and treated with intraperitoneal Dox (6 or 10 mg/kg). Cardiac function was evaluated with transthoracic echocardiography. The cardiac tissue was evaluated with immunohistochemistry and immunoblot for levels of 4-hydroxynonenal, NAD(P)H quinone oxidoreductase (NQO1), C-MYC, and dynamin-related protein 1 (DRP1) phosphorylation. Results: At 15 to 18 days, the mean ejection fraction, stroke volume, and fractional shortening were similar between the mice treated with vitamin D + Dox (10 mg/kg) vs. vehicle but significantly greater in mice treated with vitamin D + Dox (10 mg/kg) vs. Dox (10 mg/kg). Dox (10 mg/kg) increased the cardiac tissue levels of 4-hydroxynonenal, NQO1, C-MYC, and DRP1 phosphorylation at serine 616, but these increases were not observed with vitamin D + Dox (10 mg/kg). A decreased tumor volume was observed with Dox (10 mg/kg) and vitamin D + Dox (10 mg/kg). Conclusions: Vitamin D supplementation decreased Dox-induced cardiotoxicity by decreasing the reactive oxygen species and mitochondrial damage, and did not decrease the anticancer efficacy of Dox against TNBC.

## 1. Introduction

Doxorubicin (Dox) is an effective treatment for multiple cancer types, including triple negative breast cancer (TNBC). However, the use of Dox is limited because of its cardiotoxic adverse events, predominantly due to an increase in oxidative stress [1,2]. Cardiotoxicity can manifest as abnormalities in the electrical representations of heart beats as seen by an electrocardiogram, such as a prolongation in the interval between the Q wave and T wave (QT interval), decreased amplitude of QRS complexes, a decrease in left ventricular ejection fraction, and cardiomyopathy [3,4]. Dox is converted to a reactive metabolite by enzymes such as NAD(P)H quinone oxidoreductase (NQO1), producing reactive oxygen species (ROS) and changing the antioxidant balance in cells [5]. In addition, Dox increases the expression of the oncogene MYC [6]. Increased C-MYC promotes ROS production, chromosomal instability, tumor progression, and chemoresistance [1,7]. C-MYC overexpression in tumors correlates with poor prognosis and poor outcome of patients with TNBC [8,9]. The ROS scavenger dexrazoxane may limit cardiotoxicity in children receiving Dox, but its use in adults is limited [10]. Therefore, new strategies have been sought to decrease oxidative stress in individuals receiving Dox and to improve therapeutic outcomes without having detrimental effects on the antitumor activity.

TNBC is characterized by the lack of expression of the estrogen and progesterone receptors and the lack of amplification of *ERBB2* or *HER2* [11]. This cancer is difficult to treat because of the molecular heterogeneity of tumors and the lack of targetable molecules such as estrogen and progesterone receptors and *HER2* [11,12]. The typical treatment of TNBC may include a combination of surgery, radiation, and cytotoxic anthracycline chemotherapies such as Dox [13,14]. However, Dox-induced ROS, C-MYC overexpression, and C-MYC-induced ROS potentially exacerbate cardiotoxic side effects during Dox treatment of TNBC including cardiomyocyte damage, atrophy, and apoptosis [15,16].

Antioxidant use to decrease ROS during chemotherapy is typically discouraged because of theoretical concerns about decreasing drug the efficacy or by producing unknown adverse events. However, a meta-analysis of clinical trials showed that antioxidant use may have no effect or a slight beneficial effect on the results of chemotherapy [17]. Decreasing levels of Dox-induced ROS during TNBC treatment could decrease cardiotoxic adverse events, and antioxidant use during chemotherapy may benefit normal tissues and decrease the risk of cancer recurrence [17]. Vitamin D and its active metabolites have an antioxidant activity through the activation of NRF2-dependent antioxidant signaling and may inhibit tumor cell growth by downregulating C-MYC [18,19]. However, literature search showed no previous studies on the relation between systemic vitamin D supplementation and cardiotoxicity during Dox treatment of TNBC.

We hypothesized that systemic vitamin D supplementation during Dox treatment for patients who have TNBC may improve long-term outcomes after chemotherapy. The purpose of this study was to evaluate the effect of vitamin D on Dox-induced cardiotoxic adverse events in a syngeneic mouse model of TNBC.

## 2. Results

### 2.1. Dietary Vitamin D Supplementation

**Figure 1:**
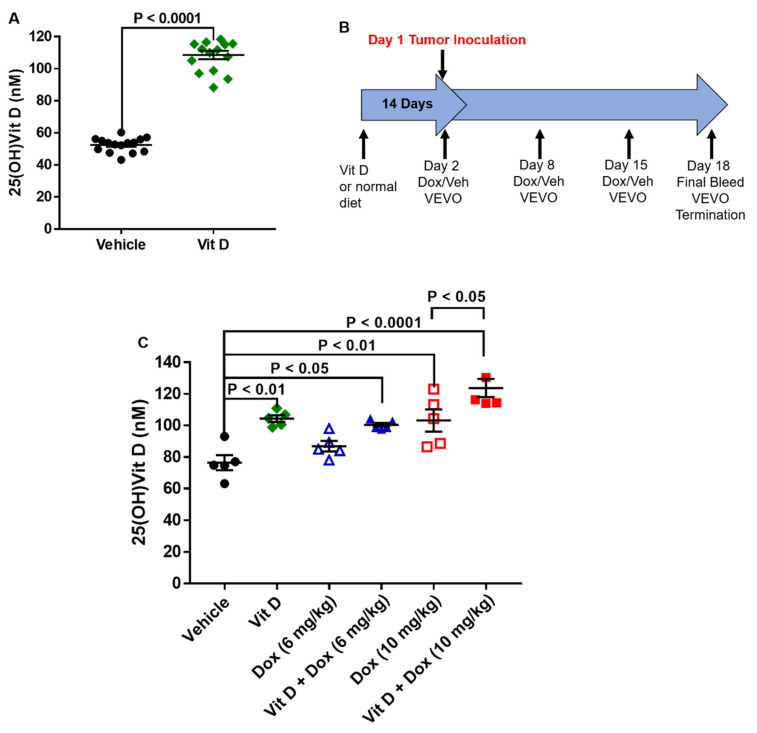
Levels of 25-hydroxyvitamin D in mice with vehicle or vitamin D-supplemented diet and doxorubicin treatment. (**A**) Levels of 25-hydroxyvitamin D (25(OH)Vit D) in the plasma collected after 14 days of 10,000 IU/kg additional vitamin D provided in the supplemented diet (15 mice per group). *p* < 0.0001. (**B**) Experimental timeline of vitamin D supplementation during doxorubicin (Dox) exposure. (**C**) Levels of 25(OH)Vit D in plasma collected 2 weeks after Dox treatment initiation (5 mice per group).

The mice were randomly assigned to receive either vehicle or vitamin D-supplemented chow for 14 days before the plasma was collected for the analysis of vitamin D concentration. Vitamin D is hydroxylated in the liver to 25-hydroxyvitamin D (25(OH)Vit D) and further hydroxylated in the kidney to 1α,25-dihydroxyvitamin D [20,21]. Analysis of the more stable 25(OH)Vit D metabolite [21,22] after 14 days of supplementation showed a two-fold increase in the mean vitamin D plasma level (vehicle, 52.5 ± 1.2 nM; vitamin D-supplemented, 108.6 ± 2.6 nM; 15 mice per group (Figure 1A). Plasma 25(OH)Vit D levels were also greater in the mice fed with supplemented vs. vehicle diet after dosing with Dox (5 mice per group) (Figure 1B,C).

### 2.2. Doxorubicin-Induced Cardiotoxicity in a Triple Negative Breast Cancer Model

**Figure 2:**
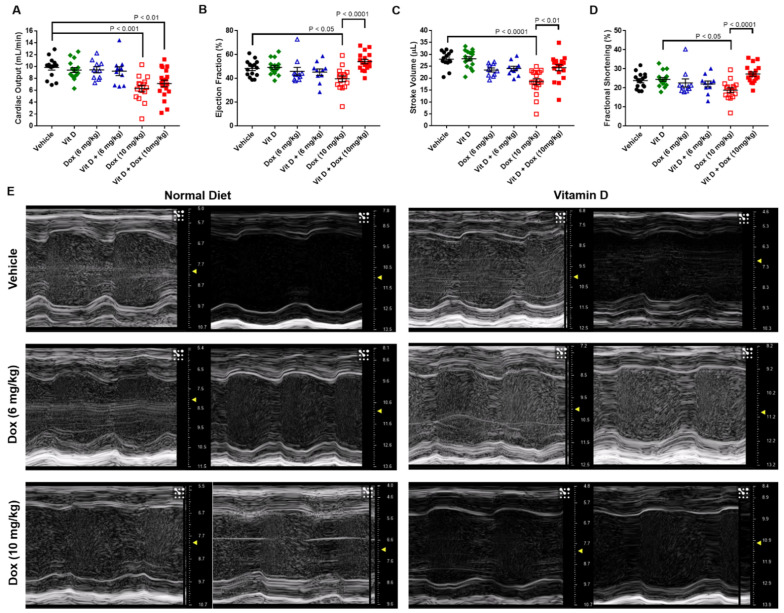
Transthoracic echocardiography of mice with vehicle or vitamin D-supplemented diet and doxorubicin treatment. (**A**) Cardiac output (mL/min). (**B**) Ejection fraction (%). (**C**) Stroke volume (µL). (**D**) Fractional shortening (%). Vehicle, 15 mice; Vit D, 16 mice; Dox (6 mg/kg) and Vit D + Dox (6 mg/kg), 10 mice each; Dox (10 mg/kg), 15 mice; Vit D + Dox (10 mg/kg), 19 mice. Videos were analyzed from day 18 for mice receiving Dox (6 mg/kg) or Vit D + Dox (6 mg/kg); all other mice were analyzed on day 15. (**E**) Still frame images extracted from echocardiography videos for two mice per group and representative of the mean of the ejection fraction.

Diet-acclimated mice were inoculated with a syngeneic mouse model of TNBC, the 4T1 murine cell line. Weekly Dox treatment started the day after tumor cell implantation, with cardiotoxicity monitored by echocardiography (Figure 1B). After two injections, totaling 14 days of Dox exposure, mice fed with vehicle (15 mice) or vitamin D-supplemented diet (16 mice) had similar mean cardiac output, ejection fraction, stroke volume, and fractional shortening without or with Dox (6 mg/kg) treatment (10 mice each) (Figure 2). Vehicle-fed mice treated with Dox (10 mg/kg, 15 mice) had decreased mean cardiac output, ejection fraction, stroke volume, and fractional shortening compared with mice that had vehicle or vitamin D supplemented diet with no Dox treatment (Table 1, Figure 2, Figure S1, Videos S1–S12). Mean cardiac output was lower in mice treated with Dox (10 mg/kg) or vitamin D + Dox (10 mg/kg, 19 mice) vs. vehicle alone. However, mean ejection fraction, stroke volume, and fractional shortening were similar between mice treated with vitamin D + Dox (10 mg/kg) vs. vehicle alone and significantly greater than observed in mice treated with Dox (10 mg/kg) (Table 1).

### 2.3. Dox-Induced Generation of Reactive Oxygen Species In Vivo

Several metabolic enzymes convert Dox to doxorubicinol, the quinone metabolite linked with cardiotoxic side effects [23]. The metabolic conversion of Dox increases ROS and alters the pools of metabolic forms of nicotinamide adenine dinucleotide (NAD) within the cell. ROS generated through Dox metabolism can oxidize unsaturated fatty acids via lipid peroxidation to form several products such as 4-hydroxynonenal (4-HNE) [24]. Administration of Dox increases 4-HNE in mice, and 4-HNE functions in several signaling pathways to induce cell death and disease [25,26,27]. Additionally, lipid peroxidation that results in cell death, known as ferroptosis, has been shown to be induced by Dox [28].

**Figure 3:**
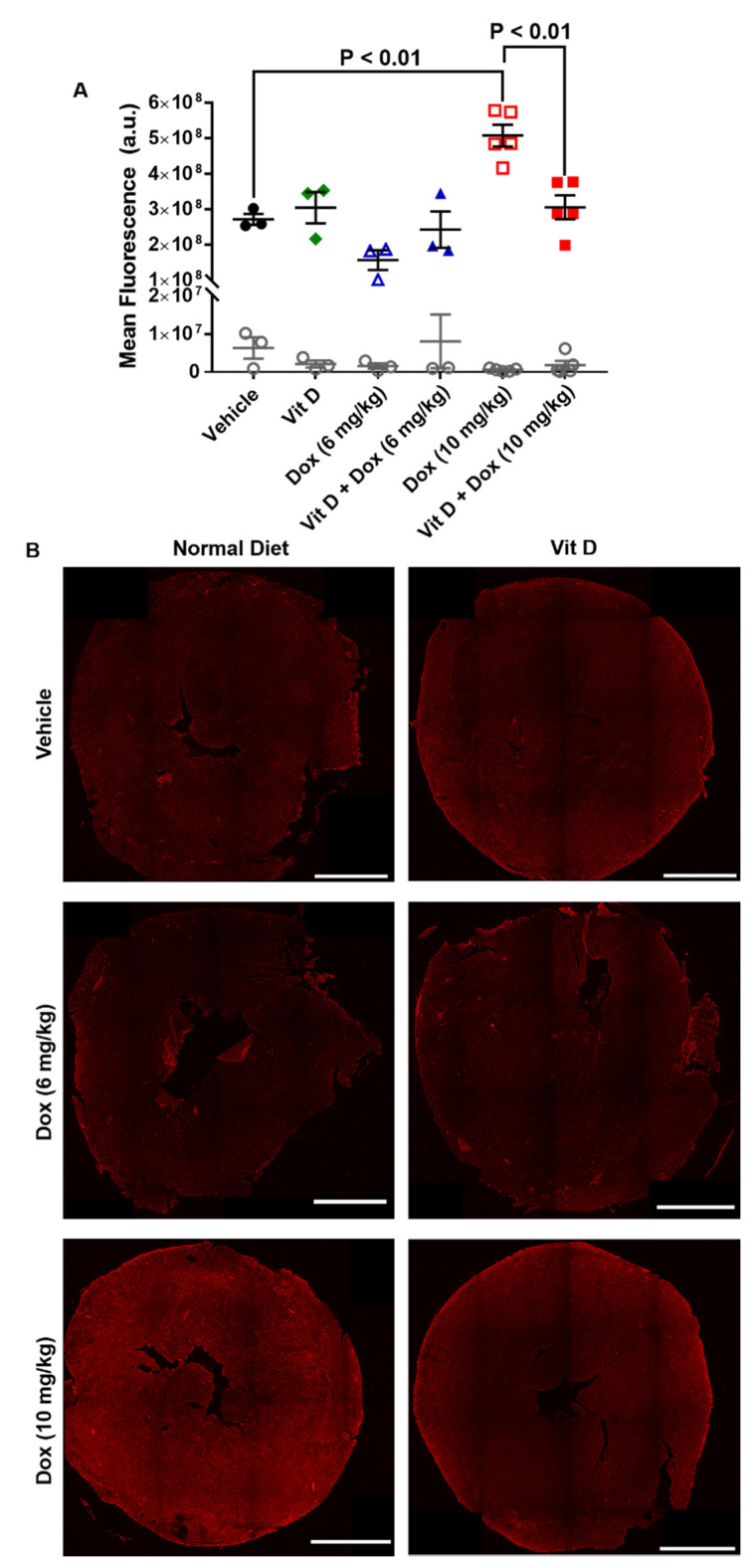
Levels of 4-hydroxynonenal in cardiac tissue of mice treated with vitamin D and doxorubicin. (**A**) Fluorescence intensity of cardiac 4-HNE. Comparison of Dox (10 mg/kg), (5.1 ± 0.3) × 10^8^ AU vs. vehicle, (2.7 ± 0.2) × 10^8^ AU; *p* < 0.01. Comparison of Dox (10 mg/kg), (5.1 ± 0.3) × 10^8^ AU vs. Vit D + Dox (10 mg/kg), (3.1 ± 0.3) × 10^8^ AU; *p* < 0.01. Comparison of Vit D, (3.0 ± 0.04) × 10^8^ AU; Dox (6 mg/kg), (1.6 ± 0.3) × 10^8^ AU; or Vit D + Dox (6 mg/kg), (2.4 ± 0.5) × 10^8^ AU vs. vehicle were nonsignificant. (**B**) Representative sections of cardiac tissue (thickness, 5 µm) showed the greatest 4-HNE (shown in red) fluorescence intensity for mice treated with Dox (10 mg/kg). Dox (10 mg/kg) and Vit D + Dox (10 mg/kg), five mice per group; all other groups, three mice per group (scale bar, 1000 µm).

Cardiac sections from mice representing the mean ejection fraction values were stained for the presence of 4-HNE (Figure 3, Figure S2). The mean fluorescence intensity of cardiac 4-HNE was significantly greater (*p* < 0.01) in mice treated with Dox (10 mg/kg, *n* = 5) (5.1 × 10^8^ ± 3.1 × 10^7^) than vehicle (*n* = 3) (2.7 × 10^8^ ± 1.6 × 10^7^) (Figure 3A), and greater with Dox (10 mg/kg) than vitamin D + Dox (10 mg/kg, *n* = 5) (3.1 × 10^8^ ± 3.3 × 10^7^) (*p* < 0.01, Figure 3A). Mean fluorescence intensity was similar between mice treated with vitamin D, Dox (6 mg/kg), or vitamin D + Dox (6 mg/kg) (*n* = 3 per group) vs. vehicle (Figure 3A). IgG staining control for 4-HNE did not show significant staining for any of the groups tested (Figure 3A). Representative images of transverse sections of mouse hearts stained for 4-HNE are shown for each group in Figure 3B. Additional images from each group are shown in Figure S2.

**Figure 4:**
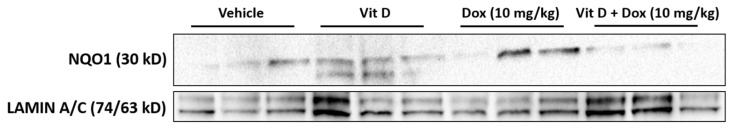
Immunoblot for NAD(P)H quinone oxidoreductase (NQO1) expression in mouse cardiac tissue. LAMIN A/C was the loading control. NQO1 expression was lower in mice treated with vitamin D + Dox (10 mg/kg) vs. Dox (10 mg/kg). Each lane represents the results for one mouse; three mice per experimental group were analyzed.

The enzyme NQO1 reduces quinones to protect cells from quinone-mediated ROS [29,30]. Immunoblot of cardiac tissue showed that NQO1 expression was induced in mice treated with Dox (10 mg/kg) (Figure 4). However, NQO1 was lower in mice treated with vitamin D + Dox (10 mg/kg) vs. Dox (10 mg/kg), consistent with decreased ROS production in mice supplemented with vitamin D (Figure 4).

### 2.4. MYC Expression from Exposure to Doxorubicin

Generation of ROS induces activation of C-MYC through exogenous addition of ROS and the generation of ROS by Dox [6,31]. An increase in C-MYC expression can induce the expression of several genes responsible for disease initiation and progression [32]. However, vitamin D can block C-MYC transcription through increased protein turnover or sequestration of β-catenin [33,34].

**Figure 5:**
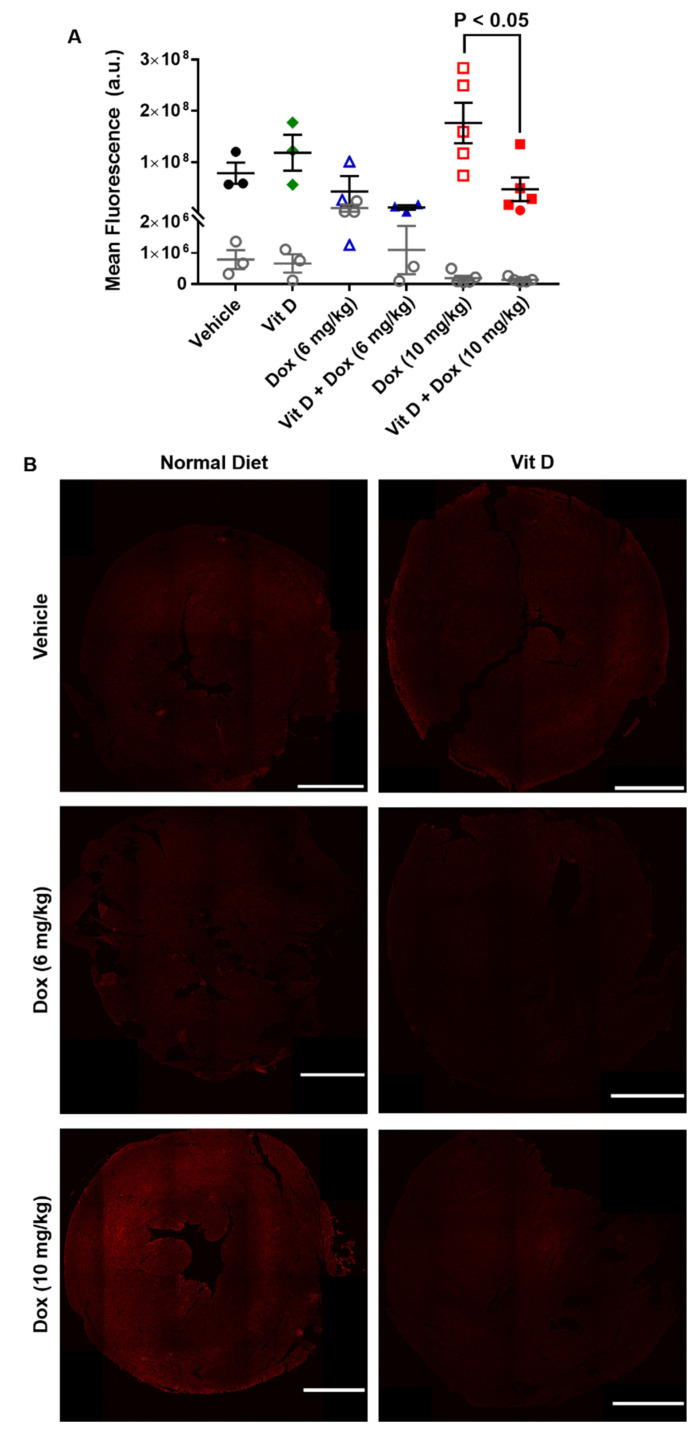
C-MYC expression in cardiac tissue of mice treated with vitamin D and doxorubicin. (**A**) Fluorescence intensity of C-MYC. Comparison of Dox (10 mg/kg), (1.8 ± 0.4) × 10^8^ AU vs. vehicle, (0.8 ± 0.2) × 10^8^ AU; *p* = 0.27. Comparison of Dox (10 mg/kg), (1.8 ± 0.4) × 10^8^ AU vs. Vit D + Dox (10 mg/kg) (0.5 ± 0.2) × 10^8^ AU; *p* < 0.05 Comparison of Vit D, (1.2 ± 0.4) × 10^8^ AU; Dox (6 mg/kg), (0.4 ± 0.3) × 10^8^ AU; or Vit D + Dox (6 mg/kg), (0.1 ± 0.04) × 10^8^ AU vs. vehicle were non-significant. Immunoglobulin G control, open gray circles. (**B**) Representative images of cardiac tissue (thickness, 5 µm) showed the greatest C-MYC (shown in red) fluorescence intensity for mice treated with Dox (10 mg/kg). Dox (10 mg/kg) and Vit D + Dox (10 mg/kg), five mice per group; all other groups, three mice per group (scale bar, 1000 µm).

The same hearts analyzed for the presence of 4-HNE were probed for the expression of C-MYC (Figure 5, Figure S3). The mean fluorescence intensity of cardiac C-MYC was similar in mice treated with Dox (10 mg/kg, *n* = 5) vs. vehicle (*n* = 3), possibly due to sample size or sample variability, but greater with Dox (10 mg/kg) (1.8 × 10^8^ ± 3.9 × 10^7^) than vitamin D + Dox (10 mg/kg, *n* = 5) (4.7 × 10^7^ ± 2.3 × 10^7^) (*p* < 0.05, Figure 5A). Mean fluorescence intensity was similar between mice treated with vitamin D, Dox (6 mg/kg), or vitamin D + Dox (6 mg/kg) (*n* = 3 per group) vs. vehicle (Figure 5A). IgG staining control for C-MYC did not show significant staining for any groups tested (Figure 5A). Representative images of transverse sections of mouse hearts stained for C-MYC are shown for each group in Figure 5B. Additional images from each group are shown in Figure S3.

### 2.5. Doxorubicin-Induced Mitochondrial Dysfunction

**Figure 6:**
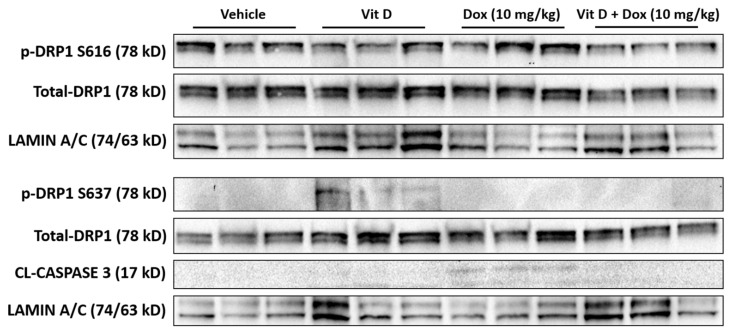
Immunoblot for dynamin-related protein 1 (DRP1) phosphorylation in mouse cardiac tissue. Phosphorylated DRP1 (p-DRP1) serine 616 (S616) was increased with Dox (10 mg/kg) but decreased with vitamin D + Dox (10 mg/kg); p-DRP1 serine 637 (S637) was increased with vitamin D but not with Dox (10 mg/kg). Cleaved Caspase 3 (CL-CASPASE 3), a marker for mitochondrial damage, was increased with Dox (10 mg/kg). Total DRP1 was included as a control, and LAMIN A/C was the loading control. Each lane represents the results for one mouse; three mice per experimental group were analyzed.

Dox treatment induces mitochondrial fission leading to cell damage or death [35]. This process is controlled, in part, through dynamin-related protein 1, DRP1 [36]. The generation of ROS leads to the activation of DRP1 through phosphorylation at S616, leading to mitochondrial localization and fission induction [36]. Conversely, the phosphorylation of DRP1 at S637 inhibits mitochondrial fission [37,38]. Immunoblot of cardiac tissue showed that phosphorylation of DRP at S616 was increased with Dox (10 mg/kg) but decreased with vitamin D + Dox (10 mg/kg) (Figure 6). In contrast, phosphorylation of DRP1 at S637 was increased with vitamin D but not with Dox (10 mg/kg). The increase in DRP1 phosphorylation at S616 coincided with a slight increase in cleaved CASPASE 3, consistent with an increase in mitochondrial damage.

### 2.6. Effects of Vitamin D Supplementation on Doxorubicin Treatment of TNBC Tumors

**Figure 7:**
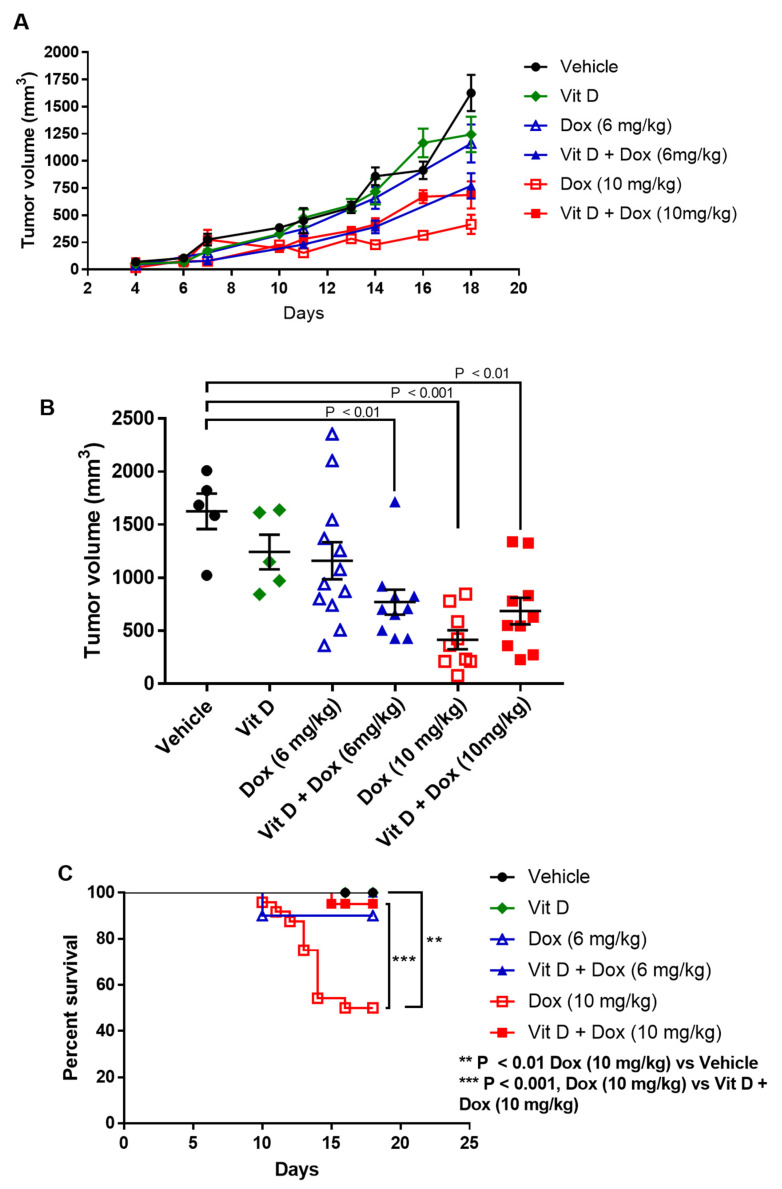
Vitamin D supplementation, doxorubicin treatment, and triple negative breast cancer tumor volume and survival. (**A**) Tumor volume over time. Number of mice per group on day 0: Vehicle or Vit D, 16 mice per group; Dox (6 mg/kg), 13 mice; Vit D + Dox (6 mg/kg), 10 mice; Dox (10 mg/kg), 24 mice; Vit D + Dox (10 mg/kg), 21 mice. (**B**) Tumor volume on day 18. Number of mice per group on day 18: Vehicle or Vit D, 5 mice per group; Dox (6 mg/kg), 12 mice; Vit D + Dox (6 mg/kg), 10 mice; Dox (10 mg/kg), 9 mice; Vit D + Dox (10 mg/kg), 10 mice. (**C**) Kaplan-Meier curves for mice treated with Vit D and Dox.

Tumor volume was analyzed over time to determine the combined effects of vitamin D and Dox. As shown in Figure 7 and Table 2, Dox treatment resulted in lower tumor volume in both 6 mg/kg and 10 mg/kg treatment groups, with 10 mg/kg providing the greatest reduction in tumor volume (Figure 7A). Further analysis of tumor volume at the termination of the experiment on day 18 is shown in Figure 7B. While vitamin D alone (*n* = 5) and Dox (6 mg/kg, *n* = 12) treatment resulted in a decrease in tumor volume, neither change reached statistical significance. However, the combination of the two treatments (*n* = 10) resulted in a significant decrease from vehicle-treated mice (*p* < 0.01). Mice treated with Dox (10 mg/kg, *n* = 9) showed a significant reduction in tumor volume compared to vehicle (*p* < 0.001). The decrease in tumor volume represents a dose-dependent effect of Dox when comparing the 6 mg/kg and 10 mg/kg concentrations (*p* < 0.01). The combination of vitamin D + Dox (10 mg/kg, *n* = 10) also resulted in a significant reduction in tumor volume compared to vehicle (*p* < 0.01).

Kaplan-Meier curves showed decreased survival for mice treated with Dox (10 mg/kg) (50% survival by day 16) vs. vehicle (100% survival at day 18) (Figure 7C). There was improved survival with Vitamin D + Dox (10 mg/kg) vs. Dox (10 mg/kg) (*p* < 0.01) such that survival was similar to vehicle. Survival was similar between mice treated with vitamin D, Dox (6 mg/kg), or vitamin D + Dox (6 mg/kg) (Figure 7C).

## 3. Discussion

The present results in a mouse TNBC model showed that low levels of vitamin D supplementation may decrease cardiotoxic adverse events during Dox treatment without decreasing Dox efficacy. Dietary vitamin D supplementation increased plasma vitamin D levels (Figure 1). Although mice exposed to Dox (10 mg/kg) had cardiotoxicity after 2 weekly injections, vitamin D supplementation prevented cardiotoxic adverse events in vivo as observed with echocardiographic parameters including ejection fraction, stroke volume, and fractional shortening (Table 1, Figure 2). Furthermore, 4-HNE and C-MYC levels were decreased and survival was significantly increased in mice receiving vitamin D + Dox (10 mg/kg) vs. Dox (10 mg/kg) alone (Figure 3, Figure 5 and Figure 7).

The increased levels of 4-HNE and expression of NQO1 were consistent with increased cardiac ROS levels induced by Dox (10 mg/kg). Vitamin D supplementation may have prevented ROS generation by Dox (10 mg/kg), evidenced by the lower 4-HNE and NQO1 levels in mice receiving vitamin D + Dox (10 mg/kg) vs. Dox (10 mg/kg) (Figure 3 and Figure 4). Suppression of NQO1 has been shown to increase sensitivity to chemotherapeutics such as Dox [39]. In addition, ROS may induce mitochondrial fission leading to cellular damage [36]. Dox treated mice showed an increase in markers indicative of mitochondrial stress such as phosphorylation of DRP1 at S616, which is a marker for the induction of mitochondrial fission [37,38]. Vitamin D treatment showed an increase in phosphorylation of DRP1 at S637, which is a marker for mitochondrial fission inhibition. Treatment with vitamin D also prevented a Dox-induced increase in C-MYC expression in cardiac tissue, consistent with previous reports showing that vitamin D can reduce C-MYC in tumor models [34]. Dox-induced C-MYC expression combined with ROS-induced oxidation of poly-unsaturated fatty acids to form 4-HNE may exacerbate cardiac dysfunction.

In the present study, vitamin D improved cardiac function without adversely affecting the decrease in TNBC tumor volume by Dox (10 mg/kg) (Figure 7). Dox-treated mice without vitamin D had greater decreases in cardiac function and survival, whereas vitamin D-supplemented mice had improved cardiac health, better survival, and slightly improved body weights (Figure S4). Although mice treated with Dox (6 mg/kg) had fewer cardiac adverse events, they also had decreased tumor volume when supplemented with vitamin D. The vitamin D supplementation used in this study (10,000 IU/kg) is equivalent to a human dose of 662 IU for a 75 kg adult, well below typical vitamin D supplement doses [40,41].

While our study indicates an involvement in the inhibition of ROS-induced mitochondrial damage, the direct mechanism of vitamin D remains elusive. However, recent studies have shown the involvement of inflammatory cytokines, including IL-1β, may exacerbate lipid peroxidation in the heart [42]. Further studies are needed in order to delineate the actions that vitamin D may have on this mechanism associated with mitochondrial damage. Additionally, at the time of TNBC diagnosis a large portion of patients present with vitamin D deficiency [43]. Ongoing studies aim to determine the role of vitamin D deficiency in the generation of ROS and ROS-induced cardiac and mitochondrial damage.

Several studies have shown the potential for vitamin D supplementation to increase the sensitivity of cancer cells to chemotherapeutic agents [44]. In addition, there are ongoing studies seeking to use vitamin D as a way to increase cancer cell exposure to Dox [45]. This study was unique in that no current study has focused on dietary vitamin D supplementation to prevent cardiotoxicity as a result of Dox treatment for TNBC in vivo.

## 4. Methods

### 4.1. Animal Care and Welfare

Female Balb.c mice (6–7 weeks old) were purchased (Charles River Laboratories, Wilmington, MA, USA). All of the animal studies followed guidelines of the National Institutes of Health (NIH) and were approved by the University of South Alabama’s Institutional Animal Care and Use Committee (protocol number 1400753 approved 29 March 2019). Michele Schuler, DVM, Ph.D., supervised all of the animal experiments and assisted when needed. All of the animals were allowed access to food and water ad libitum. The body weights were analyzed twice weekly, and the animals were observed for signs of distress or pain and were sacrificed when appropriate.

### 4.2. Dietary Vitamin D Supplementation

Rodent chow (vehicle control; vitamin D_3_, 1500 IU/kg) was obtained from the manufacturer (2019 Teklad, Envigo, Indianapolis, IN, USA) without and with an additional 10,000 IU/kg of vitamin D_3_ (referred to as vitamin D supplemented chow: total, 11,500 IU/kg) (Table 3) based on previous reports of vitamin D supplementation in mice [46]. The mice were assigned to receive vehicle or supplemented chow.

### 4.3. Blood Collection

The mice were anesthetized (isoflurane, 1–3%) until laterally recumbent, and the submandibular venous plexus was pierced with a sterile single-use lancet. For survival bleeds, blood (≤100 µL) was collected in ethylenediaminetetraacetic acid or lithium heparinized blood collection microtainer tubes (BD Microtainer, Becton Dickson, Franklin Lakes, NJ, USA) and centrifuged (5000 rpm for 5 min), and plasma was collected and stored at −80 °C for further analysis. Terminal bleeds (blood volume > 100 µL) were performed at the end of experiments immediately before euthanasia by carbon dioxide_,_ followed by cervical dislocation.

### 4.4. Vitamin D Measurement

The plasma 25-hydroxyvitamin D levels were determined with an enzyme-linked immunosorbent assay (25-Hydroxy Vitamin D^S^ EIA, AC-57SF1, Immunodiagnostic Systems, Gaithersburg, MD, USA) with all steps performed with reagents at room temperature (~23 °C) according to the instructions from the manufacturer. Biotin-labeled 25-hydroxyvitamin D was added to each sample, and the samples were added to the supplied microplate and incubated for 2 h. The plate was then washed with the provided wash buffer three times, and the enzyme conjugate was added and incubated for 30 min. The supplied substrate was added, and the reaction was allowed to proceed for 30 min and was stopped by the addition of hydrochloric acid. Absorbance at 450 nm was determined with a multimode plate reader (M1000, Tecan, Mannedorf, Switzerland), with reference absorbance determined at 650 nm. The absorbance values were normalized to the reference reading and were analyzed using a four-parameter logistic curve fit (Prism, GraphPad, San Diego, CA, USA).

### 4.5. Tumor Establishment and Doxorubicin Treatment In Vivo

The 4T1 mouse mammary tumor cell line was purchased (ATCC #CRL-2539, American Type Culture Collection, Manassas, VA, USA) within the last 12 months and passaged less than five times before implantation into the mice. Absence of rodent pathogen infection of the 4T1 cells including mycoplasma was confirmed with a polymerase chain reaction (IDEXX BioAnalytics, Columbia, MO, USA). The mice were anesthetized (isoflurane, 1–3%), and 4T1 cells (1 × 10^6^ cells in 100 µL phosphate-buffered saline (PBS) per mouse) were inoculated unilaterally in the third inguinal mammary fat pad. The next day, the mice were inoculated with intraperitoneal sterile saline (0.9% sodium chloride injection, USP) or pharmaceutical grade Dox (6 or 10 mg/kg) (Athenex, Buffalo, NY, USA). As Dox was supplied in a saline solution (2 mg/mL), the injection volume was adjusted according to the dose received and did not exceed 200 µL. The sterile saline or Dox injection was repeated after 1 week (total, two injections per mouse). The tumor length (L) and width (W) were measured twice weekly with a digital caliper and were calculated with the equation: V = L × (W^2^)/2.

### 4.6. Transthoracic Echocardiography

Mouse echocardiography was performed with an in vivo imaging system equipped with a 30 MHz transducer (Vevo 3100, FUJIFILM VisualSonics, Toronto, ON, Canada), as previously described [47,48]. The mice were anesthetized and placed on the imaging station, which contained a warming stage that detected the heart rate. The heart rates and respiratory rates were continuously monitored. Fur was removed from the neckline to the lower chest level using a topical hair removal cream. Echocardiography videos were obtained for short-axis and long-axis views. The long-axis views of the left ventricle were used to calculate the cardiac output, ejection fraction, stroke volume, and fractional shortening (a measure of left ventricular wall function) using the Vevo LAB analysis software program. Video frames were extracted (Microsoft Photos for Windows 10, Microsoft, Redmond, WA, USA), and the images presented are from mice with ejection fractions that represent the mean of the ejection fraction and were cropped for presentation.

### 4.7. Immunohistochemistry

The hearts were extracted from the euthanized mice and were transected transversely, and fixed in 10% neutral buffered formalin (inferior heart) or flash-frozen (superior heart). The fixed tissue samples were processed and embedded in paraffin, cut into 5 µm sections, and mounted on poly-lysine functionalized cover slides. The slides were processed for immunofluorescence as described previously [49]. After treatment with bovine serum albumin (BSA) 2%, the slides were incubated with primary antibodies diluted in 2% BSA in phosphate-buffered saline (PBS) and directed against C-MYC (1:200) (ab32072, Abcam, Cambridge, UK)) or 4-hydroxynonenal (4-HNE) (1:200) (ab48506, Abcam) and incubated overnight at 4 °C. Rabbit or mouse immunoglobin G (IgG) isotype control (no’s. 3900 and 5415, Cell Signaling Technology, Danvers, MA, USA) were incubated at the same concentration in µg as the primary antibodies overnight at 4 °C. The slides were washed three times for 5 min each in Tris-Buffered Saline (TBS) and were incubated with Alexa Fluor 546 goat anti-rabbit (A-11010, Invitrogen, Carlsbad, CA, USA) or Alexa Fluor 546 goat anti-mouse (A-11003, Invitrogen) for 1 h at RT. The nuclear stain Hoechst 33342 (H3570, Invitrogen) was added in the last 15 min of secondary incubation for a final dilution of 1:1000, and incubated for 15 min at RT. The slides were washed three times for 5 min each in TBS, dried, and mounted with coverslips using ProLong Gold Antifade reagent (Invitrogen). Slides were allowed to dry overnight in the dark at RT and were visualized using a Nikon A1R confocal microscope or stored at 4 °C until analysis.

### 4.8. Immunoblot

Immunoblot was performed using a lysis buffer described previously [50,51]. The lysis buffer (300–400 µL) was added to flash-frozen cardiac tissue in a dounce homogenizer, and the tissue was homogenized on ice. The samples were centrifuged at 15,000× *g* for 15 min, and the lysate was transferred to a new tube. The lysates were separated on 10% sodium dodecyl sulfate polyacrylamide electrophoresis gels (BioRad, Hercules, CA, USA) and transferred to nitrocellulose membranes. The membrane was blocked in 5% non-fat dry milk for 1 h at RT and exposed to primary antibodies diluted in 5% nonfat dry milk overnight at 4 °C. The primary antibodies were against NQO1 (ab80588, Abcam) diluted 1:5000, p-DRP1 S616 (3455, Cell Signaling) diluted 1:1000, p-DRP1 S637 (6319, Cell Signaling) diluted 1:1000, DRP1 (8570, Cell Signaling) diluted 1:1000, Cleaved Caspase-3 (9664, Cell Signaling) diluted 1:1000, and Lamin A/C (4777, Cell Signaling) diluted 1:1000. The membranes were incubated with horseradish peroxidase (HRP)-labeled secondary antibodies, either goat anti-rabbit-HRP or goat anti-mouse-HRP (Cell Signaling). HRP antibodies were detected using WesternBright Sirius (Advansta, San Jose, CA, USA). LAMIN A/C was used as a loading control because of the potential structural damage to the hearts determined by the echocardiography.

### 4.9. Statistical Analysis

Data were plotted using GraphPad Prism software and compared with *t*-test for two groups or one-way analysis of variance with Tukey’s post hoc analysis for three or more groups. The average values were reported as mean ± SEM. Fluorescent images were quantified with software (Nikon Elements, Nikon) as described previously [49]. The sum intensity was presented as the mean fluorescent intensity for each sample. Kaplan-Meier survival curves were analyzed using Mantel-Cox Log-rank test to compare the groups.

## 5. Conclusions

The present results showed that minimal or moderate dietary supplementation with vitamin D was cardioprotective and did not inhibit the chemotherapeutic activity of Dox against TNBC in mice. Further study is justified in order to evaluate the optimal dosages of vitamin D needed to improve cardiac function without decreasing Dox efficacy.

## Figures and Tables

**Table 1:** Echocardiography results.

	CO (mL/min)	EF (%)	SV (µL)	FS (%)
Vehicle	9.8 ± 0.4	48.2 ± 1.8	27.9 ± 0.9	23.9 ± 1.1
Vitamin D	9.4 ± 0.4	49.2 ± 1.6	28.1 ± 0.9	24.5 ± 1.0
Dox (6 mg/kg)	9.4 ± 0.5	45.9 ± 3.3	23.4 ± 0.8	22.6 ± 2.1
Vit D + Dox (6 mg/kg)	9.2 ± 0.7	45.2 ± 2.7	24.0 ± 1.0	22.1 ± 1.6
Dox (10 mg/kg)	6.3 ± 0.6 ***	39.6 ± 2.4 *	18.5 ± 1.2 ****	18.8 ± 1.3 ^&^
Vit D + Dox (10 mg/kg)	7.1 ± 0.6 **	54.0 ± 1.8 ^####^	28.1 ± 0.9 ^##^	27.3 ± 1.2 ^####^

CO-Cardiac Output; EF-Ejection Fraction; SV-Stroke Volume; FS-Fractional Shortening. * *p* < 0.05, ** *p* < 0.01, *** *p* < 0.001, **** *p* < 0.0001; ^##^ *p* < 0.01, ^####^ *p* < 0.0001; ^&^ *p* < 0.05; * Compared to Vehicle; ^#^ Compared to 10 mg/kg Dox; ^&^ Compared to Vitamin D.

**Table 2:** Tumor volume at day 18.

Treatment	Tumor Volume Day 18 (mm^3^)
Vehicle	1626 ± 166
Vitamin D	1244 ± 163
Dox (6 mg/kg)	1161 ± 175
Vit D + Dox (6 mg/kg)	771 ± 118 **
Dox (10 mg/kg)	415 ± 90 *** ^&^
Vit D + Dox (10 mg/kg)	687 ± 125 **

** *p* < 0.01, *** *p* < 0.001; ^&^ *p* < 0.01; ^&^ Compared with 6 mg/kg Dox.

**Table 3:** Rodent diet characteristics.

	Vehicle	Vitamin D Supplemented
Protein (%)	19.0	19.0
Fat (%)	9.0	9.0
Vitamin D_3_ (Total IU/kg)	1500	11,500

## Data Availability

The data presented in this study are contained within this article and online supplemental data at.

## Supplementary Materials

The following are available online at https://www.mdpi.com/article/10.3390/ijms22147439/s1.
