# Supplementary material for: Cytoprotective Effect of Vitamin D on Doxorubicin-Induced Cardiac Toxicity in Triple Negative Breast Cancer
Source: Int J Mol Sci. 2021 Jul 12;22(14):7439. doi: 10.3390/ijms22147439 (PMC8305038; doi:10.3390/ijms22147439)
Supplement: Supplementary file 1 [file ijms-22-07439-s001.zip › Uncropped Full gels.pdf]

Supplemental Figure 5

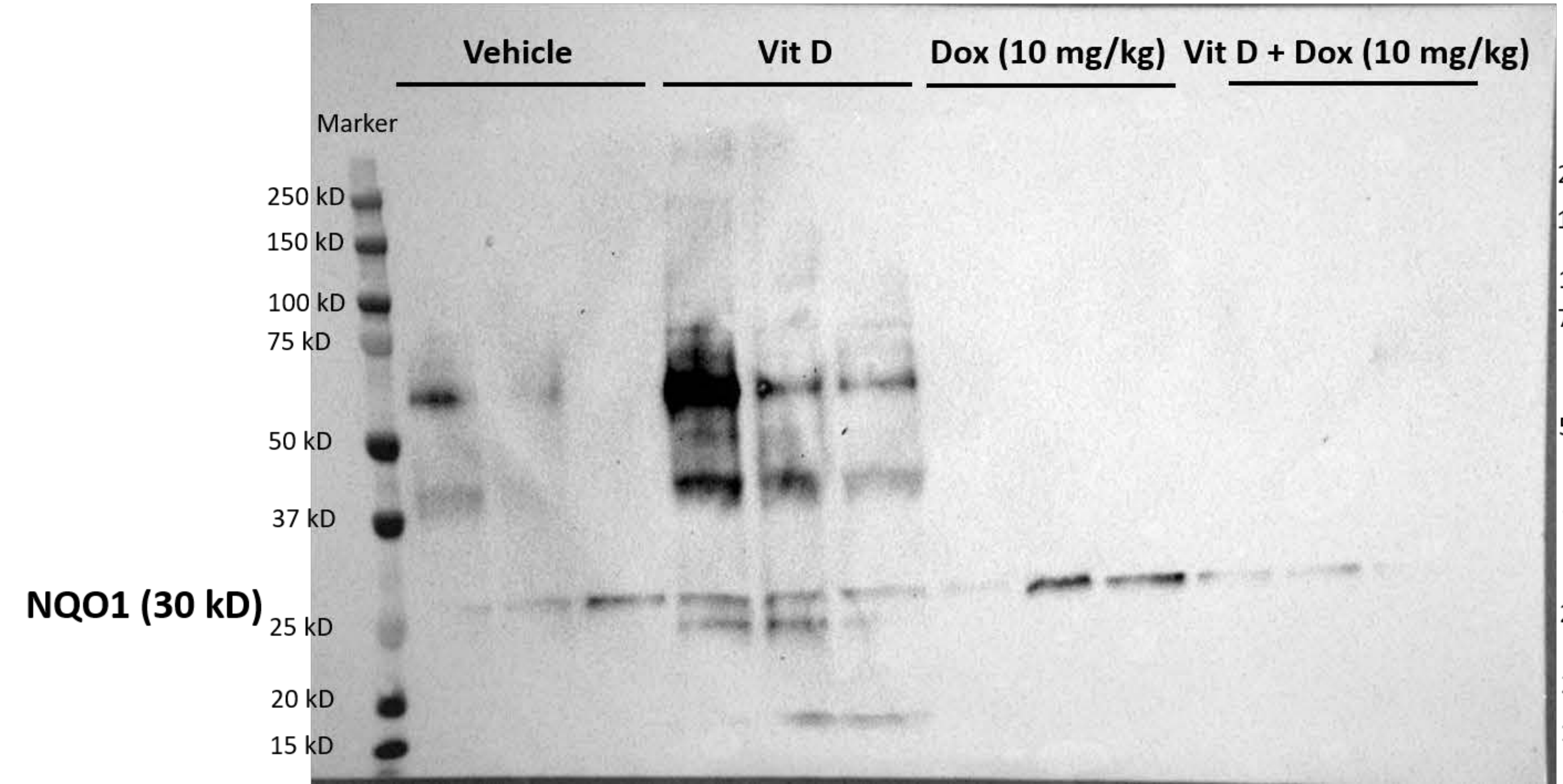

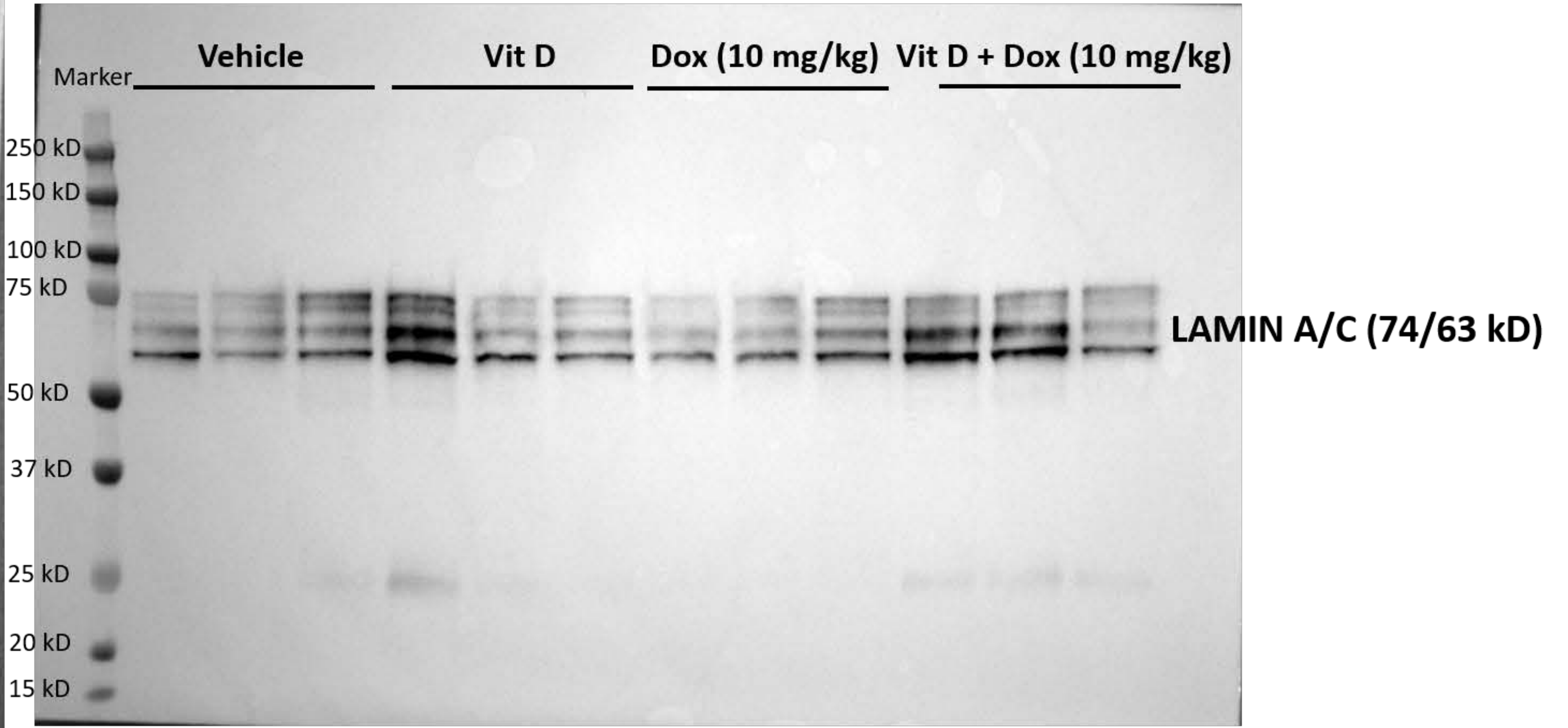

Supplemental Figure 6

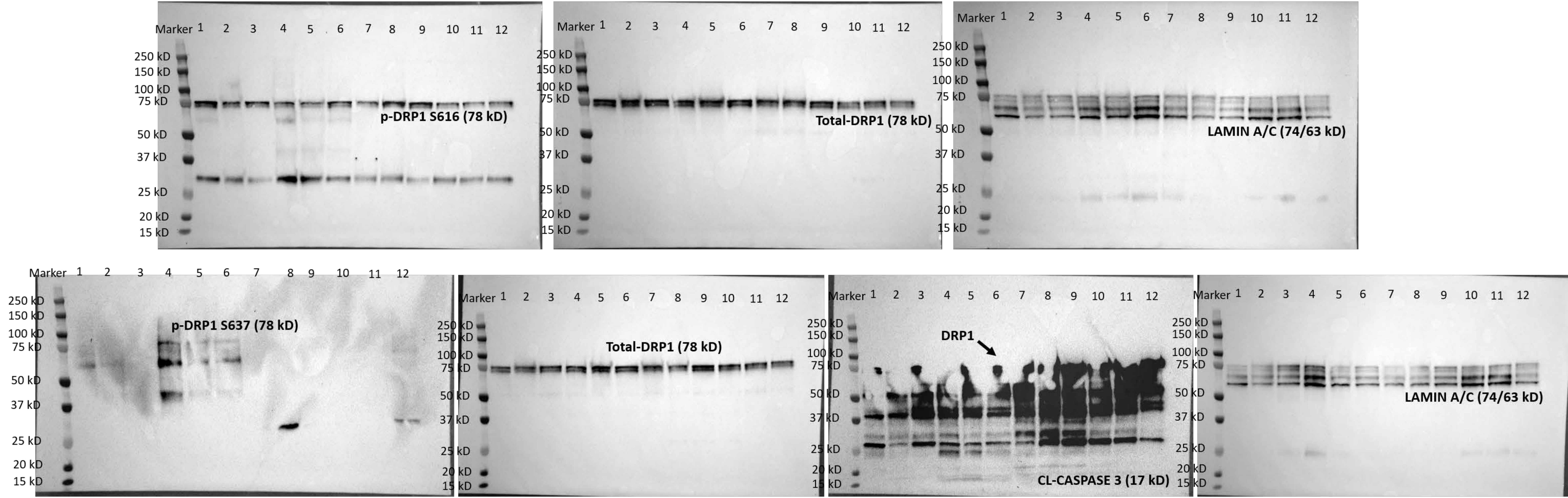

Lanes 1, 2, 3: Vehicle treated hearts; Lanes 4, 5, 6: Vit D treated hearts; Lanes 7, 8, 9: Dox (10 mg/kg) treated hearts; Lanes 10, 11, 12: Vit D + Dox (10 mg/kg) treated hearts.
