# Supplementary figures and images for: Cytoprotective Effect of Vitamin D on Doxorubicin-Induced Cardiac Toxicity in Triple Negative Breast Cancer
Source: Int J Mol Sci. 2021 Jul 12;22(14):7439. doi: 10.3390/ijms22147439 (PMC8305038; doi:10.3390/ijms22147439)

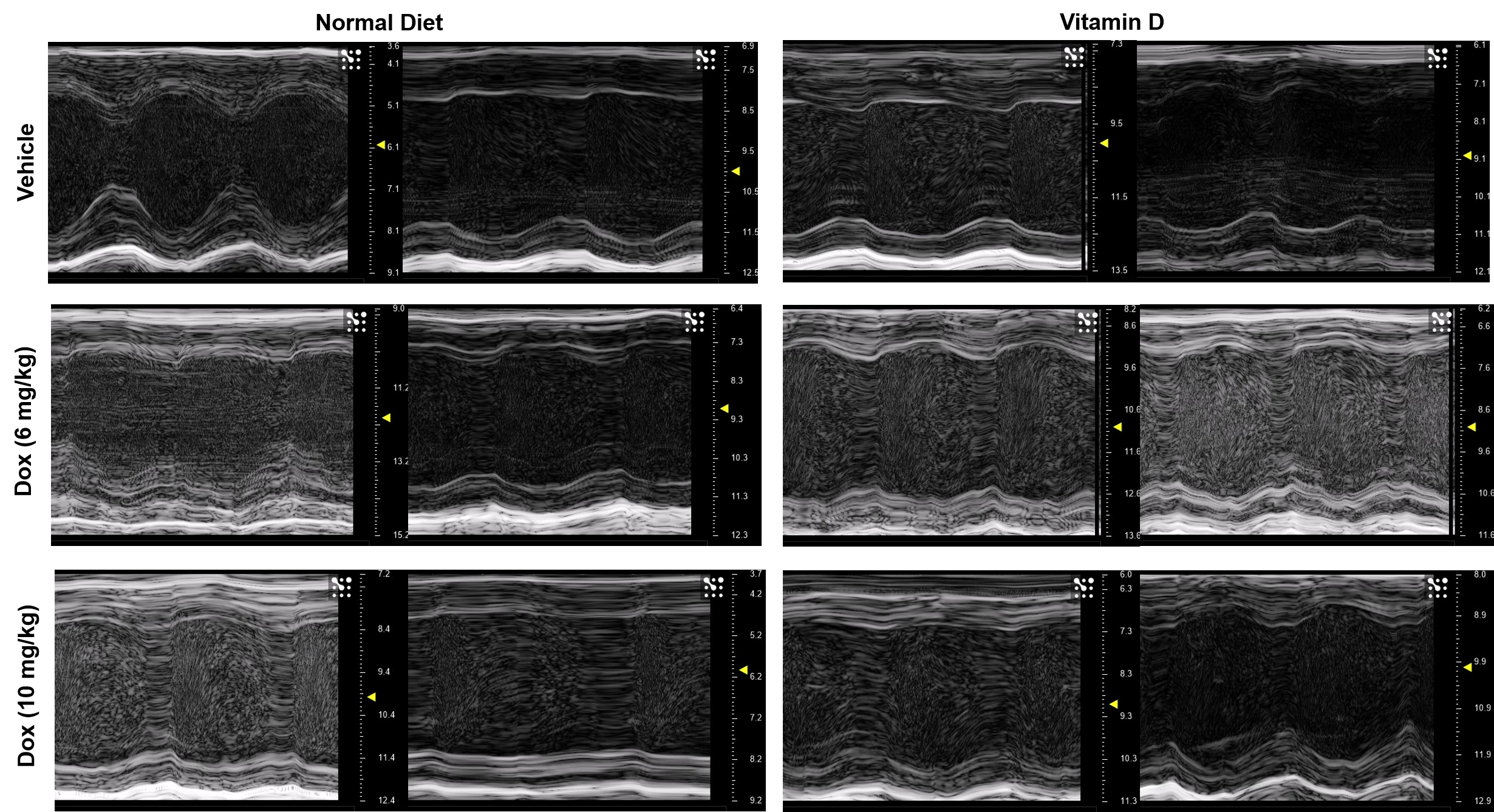

Supplement: Supplementary file 1 [file ijms-22-07439-s001.zip › Figure S1.tif]

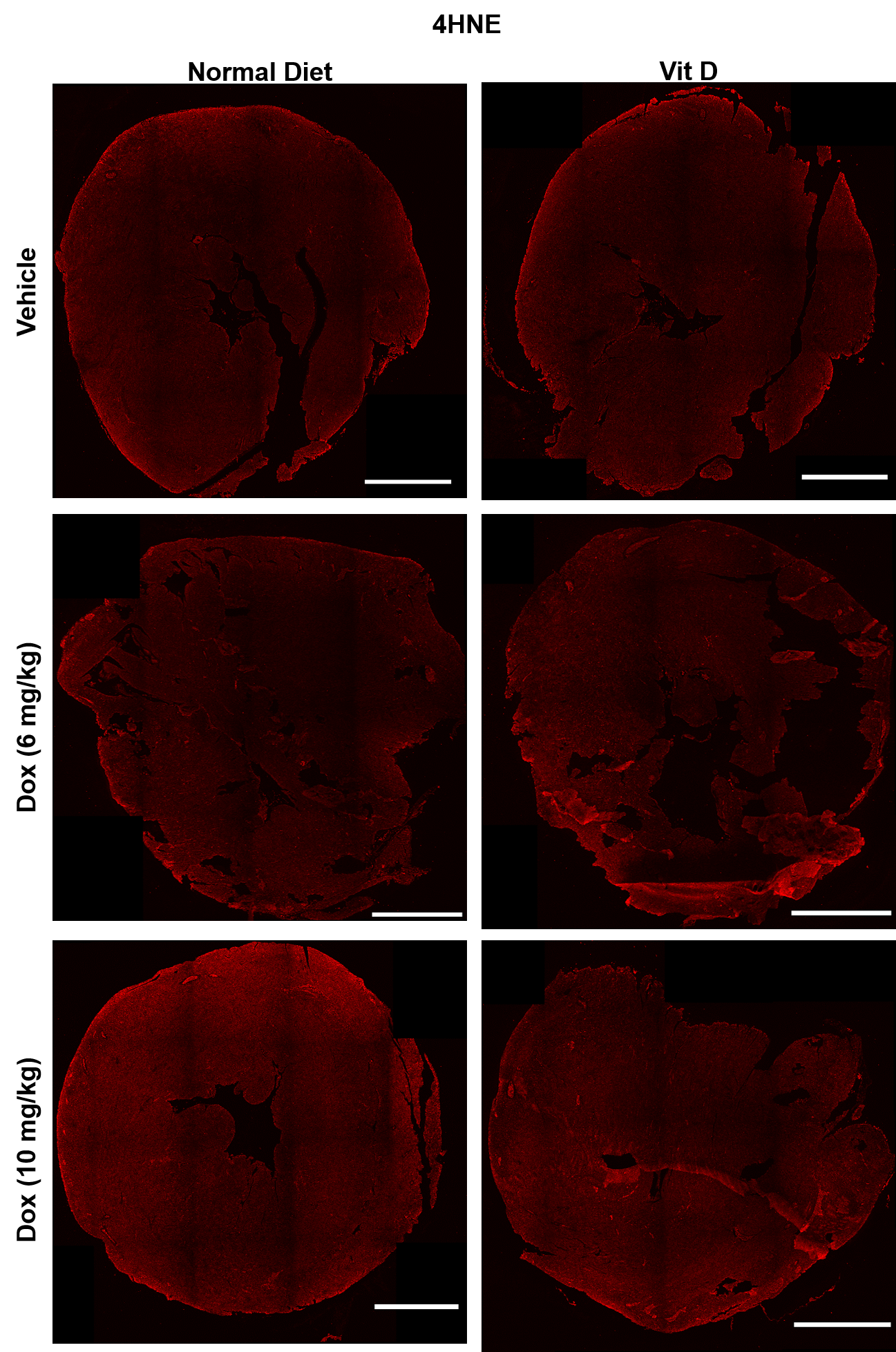

Supplement: Supplementary file 1 [file ijms-22-07439-s001.zip › Figure S2.tif]

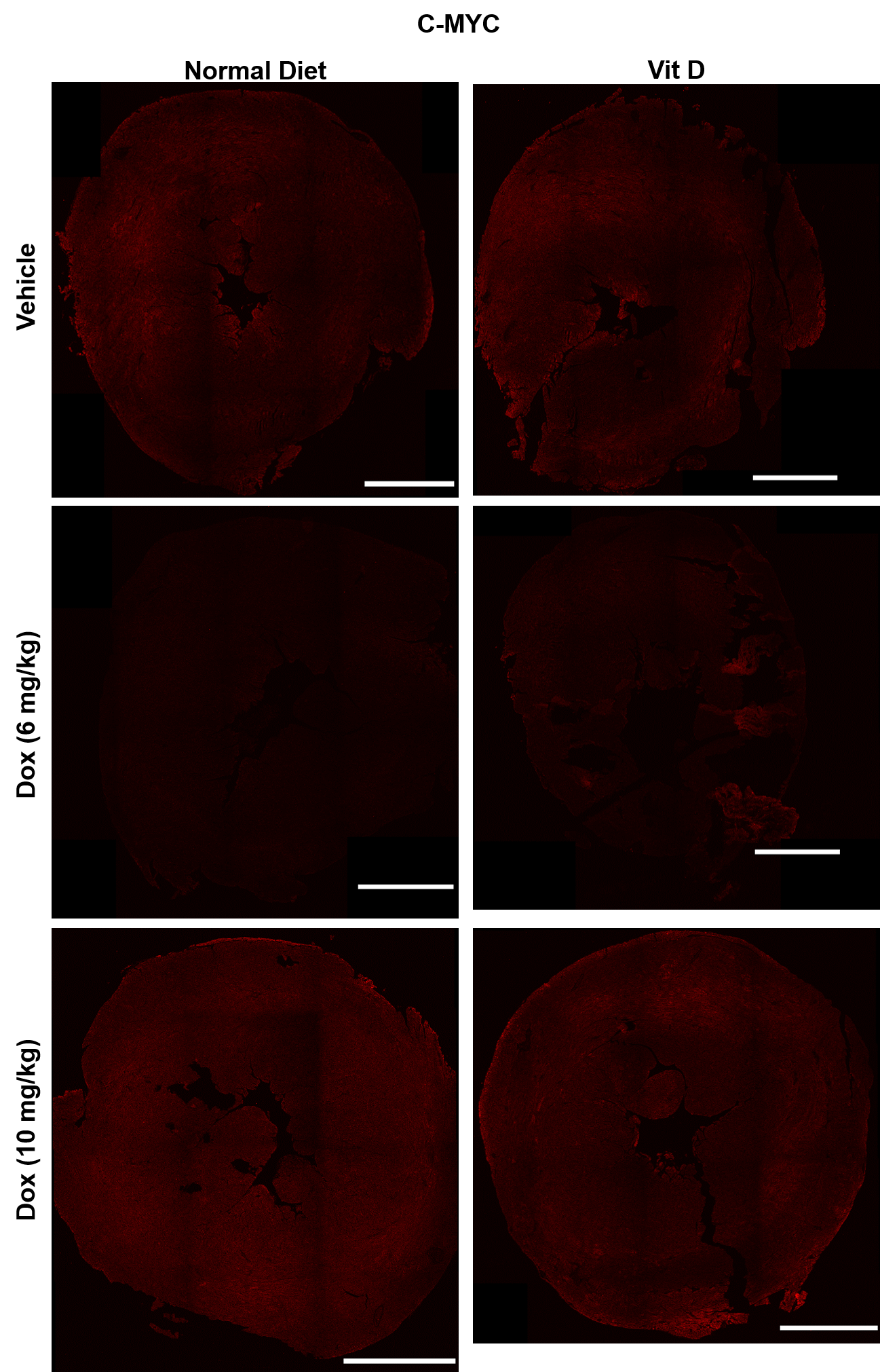

Supplement: Supplementary file 1 [file ijms-22-07439-s001.zip › Figure S3.tif]

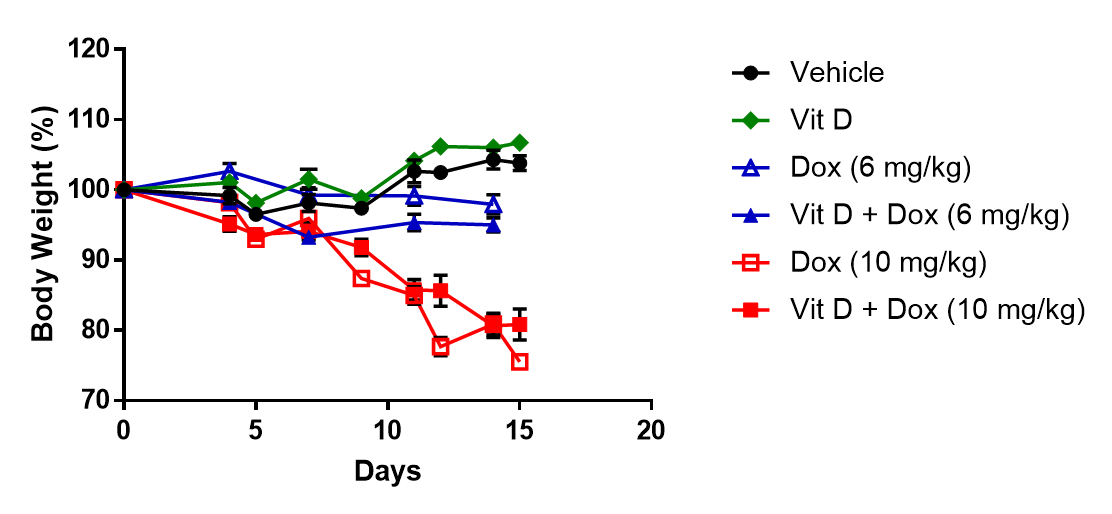

Supplement: Supplementary file 1 [file ijms-22-07439-s001.zip › Figure S4.tif]

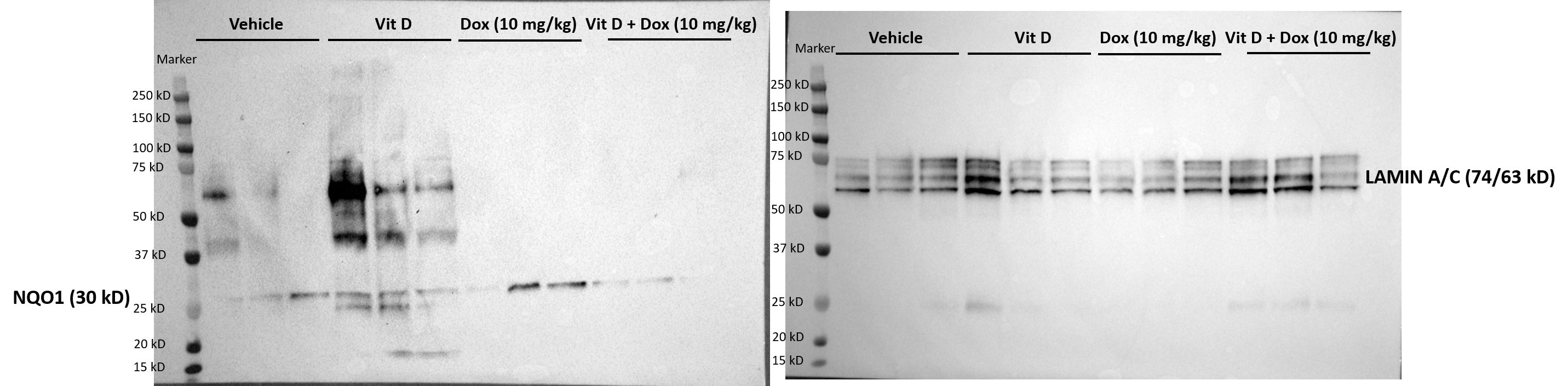

Supplement: Supplementary file 1 [file ijms-22-07439-s001.zip › Figure S5.tif]

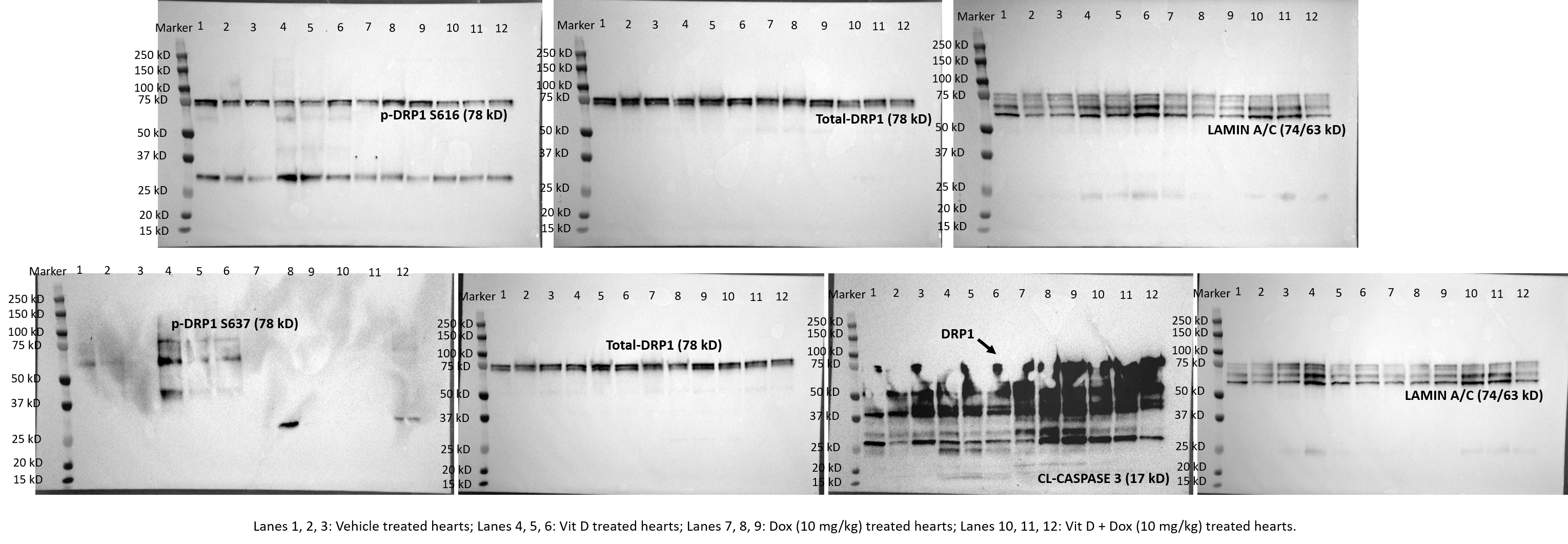

Supplement: Supplementary file 1 [file ijms-22-07439-s001.zip › Figure S6.tif]
